# Engineering Stepped Structures on Hydroxyapatite Surfaces: A Potential Strategy to Modulate Bone Marrow Mesenchymal Stem Adhesion, Spreading, and Proliferation

**DOI:** 10.3390/jfb16050165

**Published:** 2025-05-08

**Authors:** Yongmei Wang, Fang Wang, Min Gong, Lidan Chen, Yun Wang, Pu Xu, Zhu Zeng, Zuquan Hu, Jin Chen

**Affiliations:** 1 Key Laboratory of Infectious Immune and Antibody Engineering of Guizhou Province, School of Basic Medical Sciences, Guizhou Medical University, Guiyang 561113, Chinahuzuquan@gmc.edu.cn (Z.H.); 2 Key Laboratory of Biology and Medical Engineering, School of Biology and Engineering, Guizhou Medical University, Guiyang 561113, China

**Keywords:** bone marrow mesenchymal stem cells, hydroxyapatite, surface structures, stepped structures, cellular behaviors

## Abstract

Constructing the surface structures of hydroxyapatite (HA) materials is a promising strategy for orchestrating the cell behaviors of bone marrow mesenchymal stem cells (BMSCs), beneficial for advancing BMSC-based tissue repair and regenerative therapies. The majority of previous strategies have focused on fabricating artificial micro-/nano-scale geometric topographies or patterns on HA surfaces. Yet, constructing surface crystal defects has received insufficient attention and application, despite their importance as highlighted by theoretical calculations. This is largely due to the instability of crystal defects, which tend to be eliminated during crystallization. Here, given the fact that stepped structures are rich in stable crystal defects along their edges and kinks, we crafted HA dishes featuring stepped surfaces and utilized them to establish cell culture models of BMSCs. The outcomes revealed that the stepped structures markedly altered the physicochemical properties of HA surfaces and affected the cytoskeleton structures, spreading area, cell morphology, and focal adhesions of BMSCs in the cell culture model, resulting in inhibited cell adhesion. Given that YAP is a key mechanical sensitive factor, and its nuclear translocation is closely tied to cytoskeletal reorganization, the nuclear translocation efficiency of YAP has been investigated. The results showed that a changed cell adhesion could affect the nuclear translocation efficiency of YAP, which would be an important reason for the change in proliferation and differentiation ability of BMSCs. This work not only enhances the understanding of the responses of BMSCs to HA surface structures but also facilitates the design and optimization of HA materials. Moreover, our manufacturing method is facile and efficient, positioning it to potentially integrate with other processing techniques for the more effective and precise regulation of BMSCs.

## 1. Introduction

In the field of tissue engineering and regenerative medicine, bone marrow mesenchymal stem cells (BMSCs) are classified as a pivotal cell population, possessing robust proliferative capabilities and multilineage differentiation potential, providing a vital cellular reservoir for tissue regeneration [1]. Moreover, BMSCs can secrete various cytokines (e.g., VEGF, IL-4, PGE-2, and CCL2), which facilitate angiogenesis and modulate the functions of surrounding immune cells [2]. This function effectively manages the onset and progression of inflammation, substantially impacting the outcomes of tissue repair [3]. During the interaction between BMSCs and implanted materials, cell adhesion, as the initial cellular behavior, sequentially progresses through the formation of focal complexes, focal adhesions (FAs), and fibrillar adhesions [4]. Focal complexes mark the early stage of adhesion, which then mature into FAs under the contractile action of actin [5]. Eventually, these FAs evolve into more stable fibrillar adhesions, establishing a robust connection between the cytoskeleton and the extracellular matrix [6]. The surface structures of the implanted materials have been proven to exert a profound impact on cell adhesion [7]. For instance, the micro- and nano-topography of the materials’ surfaces can modify surface roughness, surface tension, and charge density and even affect the affinity for protein adsorption [8,9,10]. These alterations in the physicochemical properties of materials’ surfaces can modulate the cell adhesion process (e.g., the maturation of FAs) and generate mechanical stimulation for the cells [6,11]. Inside the cell, the maturation of FAs triggers the assembly or disassembly of F-actin, promoting the dynamic reorganization of the cytoskeleton. This process helps to regulate the contractile forces of the cytoskeleton, achieving a balance of mechanical forces within and outside the cell [12,13]. As this process unfolds, multiple mechanochemical signal transduction pathways may be activated or suppressed. For example, the surface structures of biomaterials can induce conformational changes in focal adhesion-related proteins (such as FAK, vinculin, and paxillin) [14]. These changes activate downstream signaling pathways (such as RhoA-ROCK and ERK), Ultimately, these signals are transduced into the cell nucleus through certain transcriptional coactivators, initiating the transcription of specific genes and the expression of their proteins [15]. YAP (yes1-associated protein) and MRTF-A (myocardin-related transcription factor A) are two important transcriptional co-activators that play key roles during the spreading of cells. They primarily influence cell morphology and function by modulating cytoskeletal dynamics and gene expression [16]. Numerous reports show an alteration in HA surface structures profoundly influences subsequent cell behaviors, such as migration, proliferation, differentiation, and self-renewal [17,18,19]. Therefore, designing and optimizing the surface structures of implanted materials to orchestrate the fate of BMSCs would be promising strategies for enhancing therapeutic outcomes in tissue repair and regenerative interventions [20].

Hydroxyapatite (HA), a quintessential bioactive ceramic, is extensively applied in the surface modification of bone repair and implant materials [7,21]. Post-implantation, the performance of HA materials is largely determined by their interaction with BMSCs [1]. Thus, investigating the influence of their surface structures on the behaviors of BMSCs represents a critical and burgeoning field of study [7,20]. Many techniques, such as polishing, etching, photolithography, and self-assembly, have been employed to fabricate artificial micro-/nano-scale geometric topographies or patterns on HA surfaces [22,23,24,25]. However, for crystalline bioceramic materials like hydroxyapatite, their surface physicochemical properties are influenced not only by these artificial topographies but also by their surface crystal structures [7], with surface crystal defects being a particularly notable aspect. Surface crystal defects are areas of incomplete crystallization on the surface, disrupting the original symmetry and periodicity of the surface lattice atoms, while also introducing unsaturated coordination atoms that serve as highly reactive sites [26,27,28]. Some research based on theoretical calculations indicates that these surface crystal defects on HA surfaces substantially alter the local roughness, charge density, surface tension, and the adsorption/desorption of proteins or amino acids, significantly impacting the physicochemical properties of HA surfaces [5,29,30,31,32]. Nevertheless, achieving a large number of stable crystal defects on the HA surface with ease and efficiency is always a major challenge, as surface crystal defects are unstable and can be easily eliminated during the process of minimizing the surface energy (e.g., through interface relaxation or recrystallization) [33,34].

In our prior research, inspired by the principle of oriented attachment, we introduced a viable approach to engineer stepped structures on the surfaces of micro-scale, plate-like HA particles [35,36]. These stepped structures, also known as step-terrace structures, are a classic type of surface crystal defect, with an abundance of crystal defects existing at their edges and kinks [26,34]. Compared to randomly isolated crystal defects, the crystal defects within stepped structures possess greater stability, attributed to the Ehrlich–Schwoebel barrier that exists between terraces [37,38]. Furthermore, this approach for crafting stepped structures is remarkably straightforward, avoiding the need for precise control over supersaturation, as required by traditional methods such as screw dislocation-driven growth, 2D nucleation, and epitaxial growth [39,40,41]. In particular, combining this method with vacuum-assisted filtration allows for the production of macro-scale HA dishes that have been proven suitable for cell culture applications [36]. Consequently, this approach opens up the possibility of investigating the effects of HA surface crystal defects on the cell behaviors of BMSCs.

In this work, we adopted this method to fabricate macro-scale HA dishes featuring stepped structures on their surfaces. As a control, HA dishes with smooth grains were also prepared. The physicochemical properties of these HA dishes were studied, including hydrophilicity, surface stoichiometric ratios, and protein adsorption capacity. Subsequently, BMSCs were seeded on these HA dishes, and cell adhesion was comprehensively investigated, encompassing cell morphology, cytoskeleton organization, and focal adhesion maturation. Additionally, the proliferation and differentiation capacities of the BMSCs were also analyzed, along with the transduction of certain mechanical signals, such as yes-associated protein (YAP) and myocardin-related transcription factor-A (MRTF-A). The flowchart of the entire work is displayed in Figure S1. The results indicated that stepped structures significantly altered the physicochemical properties of HA surfaces, potentially exerting profound influences on the cell behaviors of BMSCs. This work not only offers new insights for the design and optimization of HA materials but also, theoretically, enables the more effective and precise regulation of BMSCs by integrating our method with other established techniques.

## 2. Materials and Methods

### 2.1. Materials

Complete culture medium (RAXMX-90011) and alizarin red solution (ALIR-10001) were purchased from Cyagen Biosciences Inc. (Guangzhou, China). Albumin (A5010), DAPI (C0060), and rhodamine-labeled phalloidin (CA1610) were obtained from Solarbio Life Sciences Co., Ltd. (Beijing, China). Histone (C15756267) was acquired from Macklin Biochemical Technology Co., Ltd. (Shanghai, China). HA nanoparticles with a size of 200–500 nm were purchased from Macklin Biochemical Technology Co., Ltd (Shanghai, China). All other chemicals applied in this work were analytical grade without further purification.

### 2.2. Synthesis of HA Dishes

For the HA dishes with stepped structures on their surfaces, a method optimized from our previous work was applied [35,36]. In brief, 5.7 g of Ca (NO_3_)_2_·4H_2_O and 2.07 g of NH_4_H_2_PO_4_ was respectively dissolved into 400 mL of 30 mM CH_3_COONa solution to prepare two different solutions, followed by mixing and adding 0.75 mL of acetic acid. After heating at 60 °C for 12 h, the obtained CaHPO_4_·2H_2_O particles were collected, washed, and dried. Then, 0.2 g of CaHPO_4_·2H_2_O particles was resuspended in 200 mL of 1 M NaOH solution (pre-heated to 95 °C) and maintained at 95 °C for 5 min. The white HA mesocrystal particles were collected by filtration using 0.22 μm of nylon membrane and then washed in distilled water and dried at room temperature. To shape them into dishes, the HA mesocrystal particles were resuspended in distilled water, underwent a vacuum-assisted filtration process, and were dried at room temperature. Finally, the HA dishes were heated in air up to 1100 °C (at a heating rate of 5 °C min^−1^) and sintered at 1100 °C for 1 h. The obtained HA samples were named Meso-1. For the HA dishes without stepped structures on their surfaces, HA nanoparticles were used in place of HA mesocrystal particles under the same fabrication process (i.e., the vacuum-assisted filtration and the sintering process). The obtained samples were then named Nano-1.

### 2.3. Characterization

To determine the formation of the stepped structures, the microstructures of the HA dishes were observed using scanning electron micrographs (SEM, Thermos Scientific, Apreo 2C, Waltham, MA, USA). X-ray photoelectron spectra (XPS) were recorded using X-ray photoelectron spectroscopy (Thermo Fischer, ESCALAB Xi+, Waltham, MA, USA) to probe the surface elemental composition and chemical bonding states of HA, and all the binding energies were referenced to the C 1s peak at 284.8 eV. Powder X-ray diffraction (XRD) studies were performed using an X-ray diffractometer (Rigaku, Ultima IV, Tokyo, Japan) to analyze the phase and crystallinity of the HA samples. The functional groups of samples were evaluated by a Fourier-Transform Infrared Spectrometer (FTIR, Nengpu Technology Co., Ltd., iCAN 9 PLUS, Tianjin, China). Dynamic contact angle measurements were used to determine the surface hydrophilicity of the different HA dishes using a droplet shape analyser (Kruss Scientific, DSA100, Hamburg, Germany), and the images of water droplets contacting the surfaces of the samples were recorded within 0–60 s.

### 2.4. Protein Adsorption Measurement

Two protein solutions (500 μg mL^−1^) were pre-prepared by dissolving albumin or histone powders in PBS solution. Then, 100 μL of protein solution was added to the surface of each HA dish. After incubating at 37 °C for 2 h, the HA dishes were carefully washed twice with PBS solution. The concentration of protein adsorbed on the surface of each sample was measured using a BCA protein assay kit (Fdbio science, FD2001, Hangzhou, China) according to the manufacturer’s instructions. The mass of adsorbed protein per unit area of HA was calculated.

### 2.5. Culture and Identification of BMSCs

SD rats (male, 140–160 g, 5 weeks) were used to obtain BMSCs, and the procedures were approved by the Animal Ethics Committee of Guizhou Medical University (Ethical Clearance number 2100254, approval date 1 March 2021). The rats were anesthetized and sacrificed in a sterile environment. Then, bone marrow was aspirated from the femora and tibias and repeatedly rinsed with a complete culture medium. The collected cell suspension was maintained at 37 °C in a humidified 5% CO_2_ incubator for 3 days to obtain adherent cells. With a refreshing of the complete culture medium every 3 days, the adherent cells were cultured to passage 3–4 for subsequent experiments.

The biomarkers of the obtained BMSCs were identified by FITC-CD45 (561886, BD Biosciences, San Jose, CA, USA) and APC-CD29 (17-0291-82, Thermo Fisher Scientific, Waltham, MA, USA) antibody using flow cytometry (CytoFLEX Flow cytometry, Beckman Coulter, San Diego, CA, USA). The multilineage differentiation ability of BMSCs was tested by osteogenic and adipogenic induction. For the osteogenic induction, the BMSCs were cultured with an osteogenic induction medium (RAXMX-90021, Cyagen Biosciences Inc., Guangzhou, China) for 7 days, and then stained with alizarin red. For the adipogenic induction, the BMSCs were cultured with an adipogenic induction medium (RAXMX-90031, Cyagen Biosciences Inc.) for 4–7 days. The obtained cells were stained with anti-PPAR-γ (MA5-41659, Thermo Fisher Scientific) and observed using a fluorescence microscope (Nikon, i80, Tokyo, Japan).

### 2.6. Cytotoxicity Assay HA Dish Extracts

Each HA dish was washed twice with PBS and immersed into 1 mL of basal medium. After incubating at 37 °C for 48 h, the extract was obtained by filtration using a 0.22 μm Millipore filter, followed by adding 10% fetal bovine serum containing culture supplement. The BMSC suspension (200 μL, 5 × 10^4^ cells per mL) was added into each well of a 48-well plate, followed by culturing at 37 °C and 5% CO_2_ for 12 h. Then, the original culture medium of each well was replaced by 200 μL of extract obtained from each HA dish or fresh complete culture medium (set as the control group). The BMSCs were further incubated at 37 °C and 5% CO_2_ for different times (12–96 h). The cell viability of the BMSCs was determined by Cell Count Kit 8 (CCK-8, Apexbio, Houston, TX, USA), and the optical density (OD) value at 450 nm was measured using a Multiscan Spectrum (Bio Tek, Winooski, VT, USA).

### 2.7. Immunofluorescence Assay

The HA dishes (Meso-1 and Nano-1 dishes) with a 12.0 ± 0.2 mm diameter were washed twice with PBS and placed into 24-well plates. Then, 200 μL of BMSC suspension (2 × 10^5^ cells mL^−1^) was added into each well, followed by the addition of 400 μL of complete culture medium. After incubating at 37 °C and 5% CO_2_ for different times (6–96 h), the cells were sequentially washed with PBS, fixed with 4% formaldehyde, washed with PBS twice, permeabilized with 0.5% Triton X-100, and then blocked with 1% BSA solution. The collected cells were stained with rhodamine-labeled phalloidin for F-actin, DAPI for nuclei, anti-vinculin conjugated with Alexa Fluor 488 (53-9777-82, Thermo Fisher Scientific) for vinculin, anti-YAP primary antibody (sc-376830, Santa Cruz Biotechnology Inc., Santa Cruz, CA, USA) and anti-rat secondary antibody conjugated with Alexa Fluor 594 (8890, Cell Signaling Technology, Danvers, MA, USA) for YAP, anti-MRTF-A conjugated to Alexa Fluor 488 (sc-398675AF488, Santa Cruz Biotechnology Inc.) for MRTF-A, and anti-Ki67 primary antibody (13-5698-82, Thermo Fisher Scientific) and anti-mouse secondary antibody conjugated with Alexa Fluor 488 (A-11006, Thermo Fisher Scientific) for Ki67. The samples were viewed using an upright fluorescence microscope (Nikon, i80). The cell spreading areas and morphometric descriptors, including roundness, circularity, and solidity, were analyzed in Image J(Java 1.6.0) software. The roundness was mathematically calculated as 4/π × area × (major axis length)^−2^, circularity was mathematically calculated as 4π × area × (perimeter)^−2^, and solidity was mathematically calculated as area × (convex area)^−1^.

### 2.8. RNA Sequencing

The HA dishes (Meso-1 and Nano-1 dishes) with a 32.8 ± 0.3 mm diameter were washed twice with PBS and placed into 6-well plates. Then, 1.5 mL of BMSC suspension (2 × 10^5^ cells mL^−1^) was seeded onto the surfaces of HA dishes or culture plates (set as the control group), and the total number of BMSCs in each group was ensured to be 1.5 × 10^6^ cells. After 1 or 7 days of incubation, the HA dishes were carefully removed and placed into fresh 6-well plates. The BMSCs on the surfaces of the HA dishes were lysed using TRIzol reagent (15596026, Thermo Fisher Scientific) according to the manufacturer’s protocol. The collected samples were preserved on dry ice and submitted to BGI Genomics for the isolation of total RNA, library preparation, and subsequent RNA sequencing. The Kyoto Encyclopedia of Genes and Genomes (KEGG) pathway enrichment analyses were performed using the online platform of BGI Genomics (https://www.bgi.com/global accessed on 29 April 2025).

### 2.9. Statistical Analysis

Data are presented as mean ± standard deviation (SD) and were analyzed using Student’s *t*-test for two-group comparisons and a one-way analysis of variance (ANOVA) for multi-group comparisons. Statistical significance was considered at * *p* < 0.05 and ** *p* < 0.01. NS was considered to indicate no significant difference.

## 3. Results and Discussion

### 3.1. Fabrication and Characterization of HA Dishes with Stepped Structures

**Figure 1:**
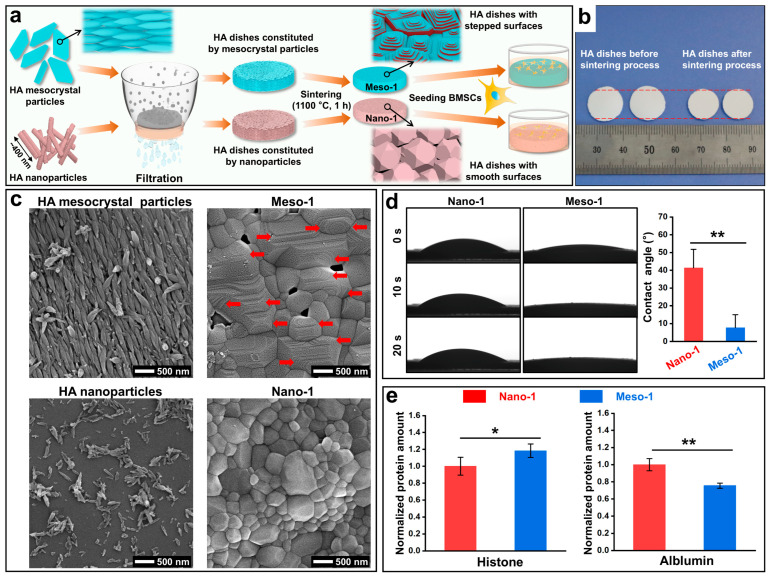
(**a**) Schematic diagram of constructing BMSC culture models using HA dishes with stepped structures exposed on their surfaces. (**b**) Digital image of HA dishes before and after the sintering process. (**c**) SEM images of HA mesocrystal particles, HA nanoparticles, Meso-1 sample, and Nano-1 sample. (**d**) The water contact angle of HA dish samples, *n* = 3. (**e**) BCA assay for proteins adsorbed on HA dish samples, *n* = 5. Values are expressed as mean ± SD, * *p* < 0.05 and ** *p* < 0.01.

To perform subsequent cell adhesion and continuous culture experiments, the HA materials were designed as macroscopic-sized dishes with stepped structures exposed on their surfaces. This stepped structure was achieved using a facile method based on oriented attachment growth [35], as shown in Figure 1a. In short, HA mesocrystal particles, prepared from CaHPO_4_·2H_2_O particles (Figure S2) through topotactic transformation [42], were dispersed in distilled water and vacuum-filtered to obtain HA dishes. Then, the HA dishes were heated at 1100 °C for 1 h to facilitate the oriented attachment process [36], including the crystallization, coarsening, and fusion of the nanoscale building units of the HA mesocrystal particles, resulting in an obvious shrinkage of the HA dishes (Figure 1b).

The SEM images (Figure 1c) clearly displayed that after the sintering process, the largely well-arranged spindle-like nanoparticles in HA mesocrystal particles disappeared and grew into much larger grains, finally resulting in a large number of stepped structures (red arrows) exposed on the surfaces of the HA dishes that were named Meso-1. According to our previous work [35,36], the interlayer step height is from 10 to 18 nm, and the terrace width is from 50 to 100 nm. Additionally, the sintering conditions (1100 °C for 1 h) could maximize the exposure of the stepped structures and result in grains with stepped structures being uniformly distributed on the surfaces of the HA dishes [36]. On the other hand, to better explore the effects of stepped structures on BMSCs, HA dishes with smooth grains (i.e., without stepped structures on their surfaces) were also prepared using HA nanoparticles (randomly arranged) instead of HA mesocrystal particles under the same fabrication process, which were named Nano-1. These HA dishes, after high-temperature sintering, would have a high crystallinity, which ensures their stability during cell culture [32,33]. The SEM images (Figure 1c) confirmed that after the sintering process, HA dishes with smooth surfaces finally formed, which was ascribed to the random arrangement of HA nanoparticles caused by vacuum filtration.

According to previous studies, stepped structures, as a kind of surface morphology during the evolution of crystals’ growth, contain a large number of crystal defects (e.g., lattice vacancies) and alter their geometric topographies [26,27,28], which usually cause some changes in their physical and chemical characteristics, such as hydrophilicity and surface stoichiometric ratios, as well as their capacity for protein adsorption [5,29,30,31,32]. Herein, contact angle measurements, XRD pattern, XPS spectra, FTIR spectra, and BCA protein assays were carried out. As shown in Figure 1d, the contact angle of the Nano-1 sample remained relatively static over time, and its value was 39.6 ± 10.35° after 20 s of dripping addition. Meanwhile, the contact angle of the Meso-1 sample was 7.7 ± 7.25° at 0 s, and it was almost zero after 10 s of dripping addition. This result implied that the generation of the stepped structures could obviously improve the hydrophilicity of the HA dishes, which might be due to the changed geometric topographies or roughness of their surfaces.

The results of the XRD patterns (Figure S3) showed that both the Nano-1 and Meso-1 samples matched the standard pattern of the HA phase (ICDD 09-0432), and there were no changes in the crystalline phase or the existence of any impurity phase. The XPS spectra (Figure S4a) displayed that the main surface elements of the Nano-1 and Meso-1 samples were C, O, Ca, and P atoms. The peaks of the C element (Figure S4b) were mainly located at 284.8 eV and 288.5 eV, and they were ascribed to adventitious carbon species coming in contact with air [43]. The O 1s peak at 530.9 eV (Figure S4c) and the Ca 2p3/2 peak at 347.1 eV (Figure S4d) corresponded to Ca−O bindings [44]; meanwhile, the P 2p1/2 peak at 133.0 eV and the P 2p3/2 peak at 133.8 eV (Figure S4e) were ascribed to the phosphorus bound to oxygen in PO_4_^−3^ groups and the P−O bindings in the calcium phosphate phase, respectively [44]. The FTIR spectra (Figure S5) showed that of the triplet peaks observed between 500 and 650 cm⁻^1^, 550 cm⁻^1^ and 605 cm⁻^1^ belonged to the P–O bending vibrations, and 638 cm⁻^1^ belonged to O–H stretching vibration, and the sharp peak at 3580 cm⁻^1^ (O–H stretching vibration) were characteristic of HA [45]. The results of the FTIR spectra, XRD patterns, and XPS spectra demonstrated that both the Nano-1 and Meso-1 samples were pure and highly crystallized HA phases without the introduction of impurities. However, there were differences in the Ca/P ratio of the HA dishes calculated by XPS spectra, with values of 1.57 for the Nano-1 sample and 1.27 for the Meso-1 sample. This suggested that, compared to the Meso-1 sample, the surface Ca/P ratio of the Nano-1 sample was closer to that of the bulk HA materials. This result indicated that the exposure of stepped structures was likely to lead to the formation of Ca^2+^ vacancies on the surfaces of HA dishes, thereby altering the surface stoichiometric ratios.

For the assessment of protein adsorption capacity, a BCA protein assay was performed to determine adsorptions of histone (globular shape, positively charged, ~7 nm diameter) and albumin (globular shape, negatively charged, ~8 nm diameter). As shown in Figure 1e, the amount of adsorbed histone in the Meso-1 sample was higher than that in the Nano-1 sample, while the amount of adsorbed albumin in the Meso-1 sample was lower than that in the Nano-1 sample. This result indicated that the HA dishes with stepped structures tended to adsorb positively charged proteins rather than negatively charged proteins, which was possibly attributed to the increased local surface electronegativity caused by the presence of Ca^2+^ vacancies on the stepped structures.

### 3.2. The Effects of Stepped Structures on the Cell Morphology and Cytoskeleton of BMSCs

Herein, BMSCs were harvested from the femoral bone marrow of SD rats using the whole bone marrow adherence method [46]. The results of the flow cytometry showed that more than 98% of passage 2 and passage 5 cells were CD45-negative, and more than 99% of cells were CD29-positive (Figure S6a), confirming that the obtained cells possessed the feature markers of BMSCs. Thus, the passage 3–4 cells were applied for subsequent experiments. In addition, the results of alizarin red staining and the PPAR-γ immunofluorescence staining indicated that the passage 3–4 cells possessed the capacity of osteogenic (Figure S6b) or adipogenic (Figure S6c) differentiation under osteogenic or adipogenic induction conditions, suggesting the multiple differentiation potential of the cells.

**Figure 2:**
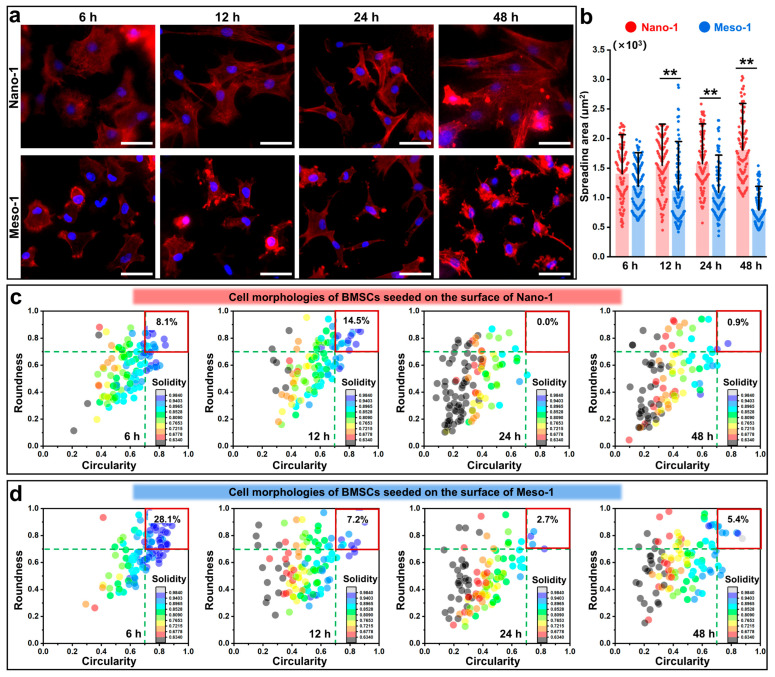
(**a**) Fluorescence microscope images of BMSCs cultured on HA dish samples for different times (nuclei were stained blue, F-actin was stained red, scale bar = 50 μm). (**b**) The cell spreading area of BMSCs, *n* = 110, ** *p* < 0.01. The cell morphology analysis of BMSCs cultured on (**c**) the Nano-1 and (**d**) Meso-1 samples, *n* = 110.

To evaluate the cell adhesion abilities of BMSCs in response to the stepped structures, BMSCs were seeded on different HA dish samples for different times (6–48 h), and the F-actin cytoskeletons were stained. As shown in Figure 2a, the cells adhered stably to the surface of the Nano-1 sample and Meso-1 sample during the whole culture process. At the early stage of adhesion (6 h), the cells could stably adhere to both the Nano-1 and Meso-1 samples and presented a rounded or polygonal shape. However, with the extension of the culture time (12–48 h), the cellular morphological changes between these two groups were significantly different. The cells of the Nano-1 group gradually spread and generated more filopodia and lamellipodia, leading to final morphologies with an elongated and irregularly stretched shape. Meanwhile, the cells of the Meso-1 group showed restricted spreading, with fewer filopodia and lamellipodia, resulting in roundish and less irregular shapes. On the other hand, the statistical results of cell areas (Figure 2b) also indicate that the spreading of BMSCs was inhibited by stepped structures. BMSCs gradually increased their cell area on the HA dishes with smooth surfaces (i.e., the Nano-1 group), while decreasing their cell area on the HA dishes with stepped surfaces (i.e., Meso-1 group). In other words, the stepped structures of HA surfaces could affect the process of cytoskeleton reorganization in BMSCs.

To further detail the effects of the stepped structures on the cell morphologies of BMSCs, shape descriptor parameters were evaluated to identify these cellular morphologies, including roundness, circularity, and solidity. According to previous reports, cells featuring values of roundness and circularity between 0.7 and 1 were considered round, whereas cells exhibiting values below 0.7 for these two parameters were deemed elongated [47,48,49]. Concurrently, solidity was utilized to estimate membrane protrusions, with lower values indicating more irregular boundaries (i.e., cells with bigger or more membrane protrusions). The distributions of these three parameters at different time points are shown in Figure 2c,d. At the early stage of adhesion (6 h), 8.1% of cells in the Nano-1 group featured values of circularity and roundness between 0.7 and 1, meanwhile, 28.1% of cells in the Meso-1 group showed values in the same range. After 48 h of culturing, only 0.9% of cells in the Nano-1 group showed circularity and roundness > 0.7; meanwhile, 5.4% of cells in the Meso-1 group showed values in the same range. On the other hand, with an extension in the culture time, the solidity values of the cells in both groups showed a significant decrease compared to their values at 6 h. This result indicated that the BMSCs gradually changed to elongated shapes and showed more irregular boundaries. Moreover, after 48 h of cultivation, the statistical results of roundness, circularity, and solidity (Figure S7) revealed that the Meso-1 group demonstrated higher values in these parameters compared to the Nano-1 group, suggesting that the BMSCs adhered to the Meso-1 sample displayed a lower degree of elongation and a lesser number of membrane protrusions, as compared to those in the Nano-1 group. Therefore, the above results indicated the adhesion of BMSCs was influenced by the stepped structures exposed on the surfaces of the HA dishes, leading to constrained spreading areas, restricted elongation, and diminished membrane protrusions.

It should be pointed out that after the sintering process, the two types of HA dishes were pure and highly crystalline phases of HA (Figure S3), devoid of any impurities or toxic substances, despite being crafted from distinct HA particle sources (the Meso-1 dishes utilized mesocrystal particles, while the Nano-1 dishes employed nanoparticles). The results of the CCK-8 assay (Figure S8) confirmed that the BMSCs cultured with extracts from different HA dishes displayed a good cell viability and proliferation. Thus, the aforementioned phenomenon of cell adhesion was more likely due to alterations in physicochemical properties (such as hydrophilicity, surface stoichiometric ratios, and protein adsorption capacity) caused by the exposure of stepped structures, rather than the release of some soluble substances (e.g., ions) that could affect the culture medium.

### 3.3. The Effects of Stepped Structures on the FAs of BMSCs

**Figure 3:**
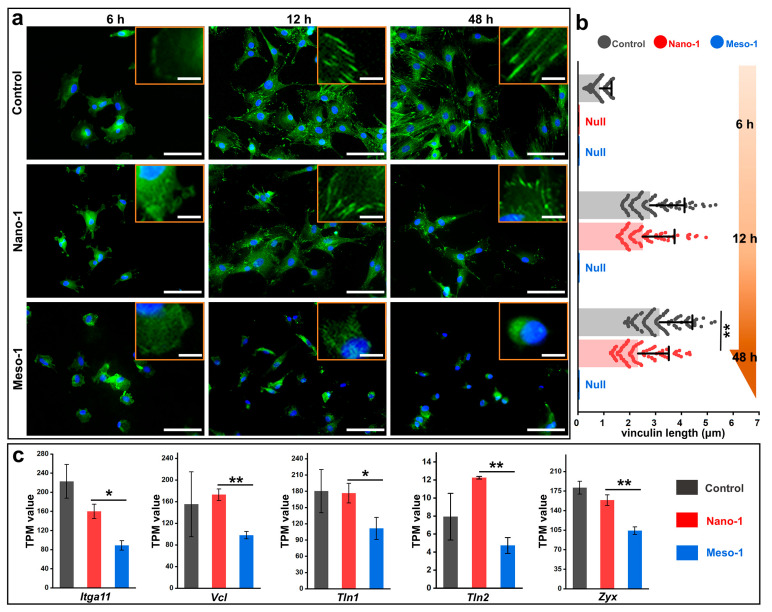
(**a**) Fluorescence microscope images of BMSCs cultured on plate or HA dish samples for different times (nuclei were stained blue, vinculin was stained green, scale bar = 80 μm); higher-magnification images are shown in the top right corner (scale bar = 10 μm). (**b**) The length of FAs, *n* = 100. (**c**) The TPM values of some genes associated with cell adhesion in BMSCs, harvested 24 h post-seeding, *n* = 3. Values are expressed as mean ± SD, * *p* < 0.05 and ** *p* < 0.01.

FAs act as a vital bridge between integrin–ECM interaction and the cytoskeleton, with their formation and maturation being crucial not only for the process of cellular adhesion but also for the subsequent mechanotransduction [6,11]. Thus, to detail the effects of stepped structures on the cell adhesion of BMSCs, the formation and maturation of FAs were characterized by the immunofluorescence staining of vinculin, a key component of FAs. In addition, the BMSCs cultured on the plates were also studied and set as the control group. As shown in Figure 3a, the BMSCs cultured on plates exhibited distinct FAs appearing as punctate dots, and with the extension in culture time (6–48 h), these punctate dots gradually elongated, indicating the maturation of the FAs. The BMSCs in the Nano-1 group also showed a clear maturation of FAs, although their maturation was slightly less robust than that observed in the control group. However, few punctate dots were observed in the BMSCs of the Meso-1 group, suggesting that the stepped structures of HA dishes could limit the formation and maturation of FAs. To better quantify the FAs, the length of vinculin was also measured and statistically analyzed. Figure 3b depicts that after adhesion stabilization, the vinculin length of the BMSCs in the control and Nano-1 groups reached micrometer scales, which were significantly longer than that of the BMSCs (their vinculin length was too short to be measured) in the Meso-1 group. This finding is consistent with the observation that the spreading of BMSCs was restricted by the stepped structures on the HA surfaces (Figure 2a,b).

To gain more insight into the formation of FAs, RNA sequencing was applied, and transcripts per million (TPM) values were utilized to assess the expression levels of some genes. As depicted in Figure 3c, some adhesion-related molecules with varying expression levels were identified, including integrin α11 (encoded by *Itga11*), vinculin (encoded by *Vcl*), talin 1 (encoded by *Tln1*), talin 2 (encoded by *Tln2*), and zyxin (encoded by *Zyx*). The TPM values of these genes of the BMSCs in the Meso-1 group were significantly lower than those of the BMSCs in the Nano-1 group, suggesting that the stepped structures could reduce the expression levels of various adhesion-related protein molecules, with vinculin being just one representative example among them. Thus, it is reasonable to believe that the inhibition of FAs’ development caused by stepped structures might be responsible for the restricted adhesion of BMSCs in the Meso-1 group.

### 3.4. The Effects of Stepped Structures on the Localization of YAP and MRTF-A

**Figure 4:**
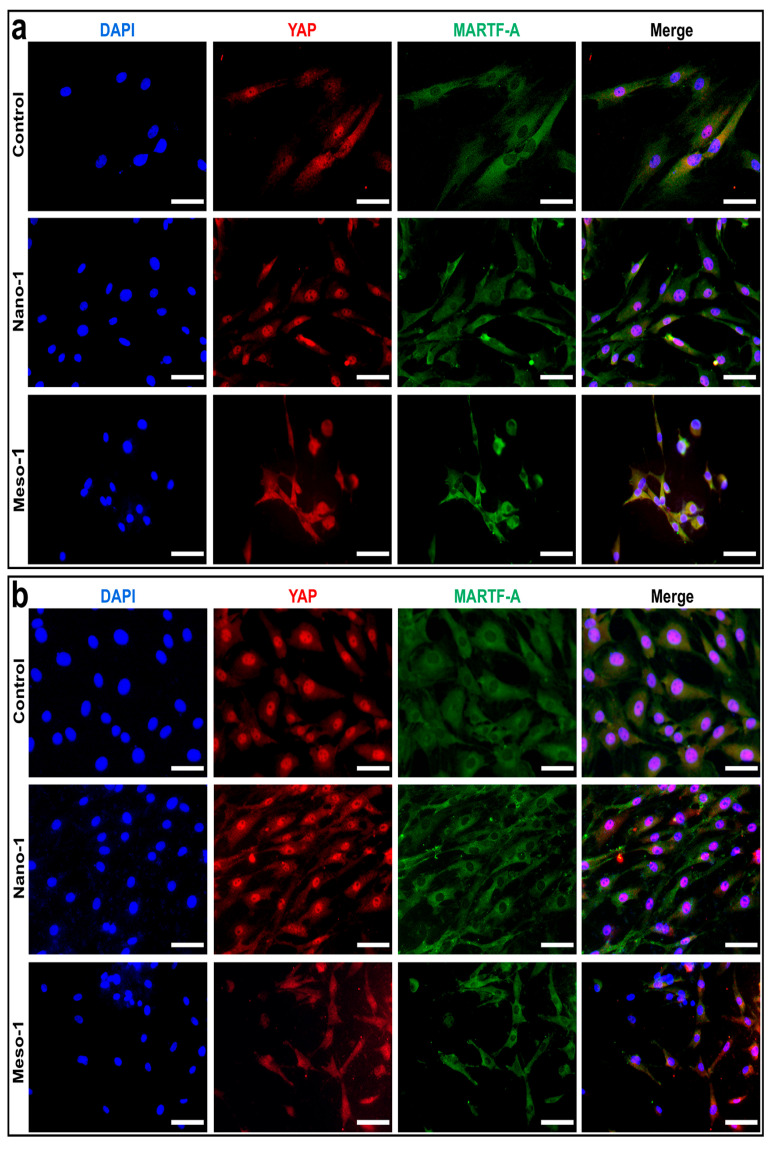
Fluorescence microscope images of BMSCs cultured on plate or HA dish samples for (**a**) 48 h and (**b**) 96 h. Nuclei were stained blue, YAP was stained red, and MRTF-A was stained green. Scale bar = 50 μm.

During the dynamic cytoskeleton reorganization of cells to balance internal forces with external mechanical forces, some mechanosensitive signaling molecules can be activated and undergo nuclear translocation, thereby regulating the expression of downstream genes and ultimately significantly affecting the functions and destiny of the cells [50,51]. Herein, the nuclear localization of YAP and MRTF-A, two transcriptional co-activators known to be dynamically regulated during the F-actin remodeling process [52,53,54], was examined. As shown in Figure 4, there was a notable accumulation of YAP within the nucleus in both the control group and Nano-1 groups, indicating that the BMSCs cultured on the smooth HA surfaces could possess a comparable nuclear translocation of YAP as those cultured on plates. Meanwhile, for the BMSCs in the Meso-1 group, the distribution of YAP between the cytoplasm and the nucleus was quite similar, without a visible accumulation of YAP in the nucleus. This lack of nuclear accumulation implied that the exposure of stepped structures on the HA surface negatively impacted the nuclear translocation of YAP. For the results of MRTF-A staining, BMSCs from all groups showed similar outcomes across different culture times (48–96 h). There was no evident nuclear accumulation of MRTF-A, whether in the control and Nano-1 group, where the BMSCs were well spread, or in the Meso-1 group, where the BMSCs exhibited restricted adhesion.

The YAP and MRTF-A staining results implied that the exposure of stepped structures on the HA surfaces mainly affected the distribution of YAP rather than MRTF-A, which was an interesting phenomenon. Although many underlying molecular mechanisms still require further investigation, the above results showed that the stepped structures on HA surfaces could alter the mechanical stimulation for BMSCs by limiting the formation and maturation of FAs, as well as restricting their cell adhesion, thereby affecting the nuclear translocation of YAP, which might have profound effects on subsequent cell behaviors.

### 3.5. The Effects of Stepped Structures on the Cell Proliferation of BMSCs

**Figure 5:**
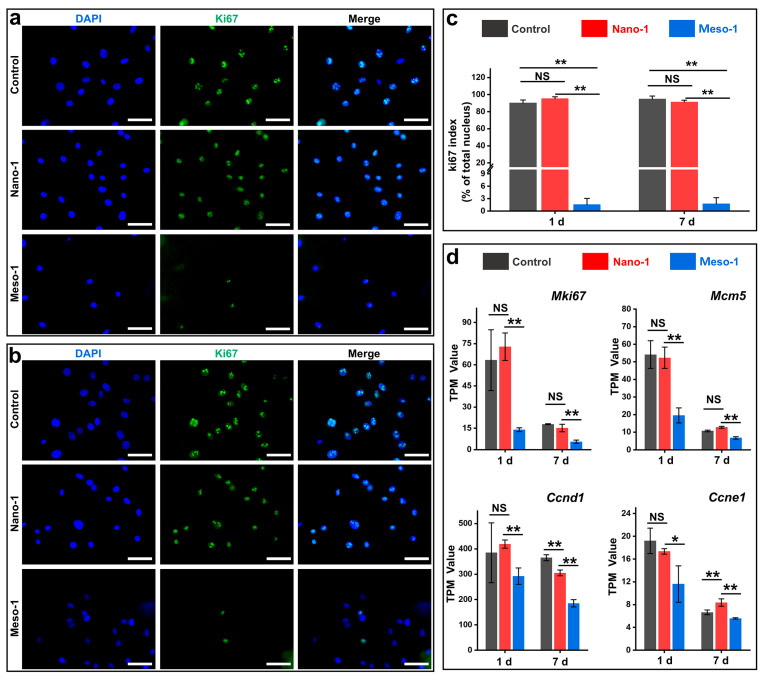
Fluorescence microscope images of BMSCs cultured on plate or HA dish samples for (**a**) 24 h and (**b**) 48 h. Nuclei were stained blue, and ki67 was stained green. Scale bar = 50 μm. (**c**) The ki67 index of BMSCs, *n* = 3. (**d**) The TPM values of genes related to cell proliferation, *n* = 3. Values are expressed as mean ± SD, * *p* < 0.05, ** *p* < 0.01, and NS represents no significant difference.

Given that YAP nuclear localization is closely associated with cell proliferation [52], the potential effects of stepped structures on the cell proliferation of BMSCs were assessed. To this end, Ki67 (a nuclear marker of cell proliferation [55]) staining was performed, and the corresponding Ki67 index (the ratio of Ki67-positive nuclei to total nuclei) was also calculated. As depicted in Figure 5a–c, after being cultured for 24 to 48 h, the BMSCs in the Nano-1 group exhibited a good proliferative capacity, with a Ki67 index exceeding 80%, which was close to the results of the control group (i.e., BMSCs cultured on plates). However, in the Meso-1 group, only a minority of BMSCs was Ki67-positive, a rate markedly lower than that of the Nano-1 group, and there was almost no visible increase in its Ki67 index even with an extension in the culture time. This result suggests that the stepped structures exposed on the HA surface could inhibit the proliferation of BMSCs.

To gain more insight into the effects of stepped structures on cell proliferation, RNA sequencing was utilized to analyze expression levels of some genes crucial for cell proliferation, including *Mki67*, *Mcm5*, *Ccnd1*, and *Ccne1*, which encode Ki67, minichromosome maintenance complex component 5, cyclin D1, and cyclin E1, respectively. As shown in Figure 5d, the TPM values of these genes in the Nano-1 group were close to those of the control group, indicating that BMSCs cultured on the HA dishes with smooth surfaces exhibited robust proliferative capacities. Compared to the Nano-1 group, the Meso-1 group exhibited considerably lower TPM values for these genes on both day 1 and day 7, implying that the proliferation of BMSCs cultured on stepped surfaces was markedly hindered. These findings were consistent with the results of the immunofluorescence staining (Figure 5a–c), which show a strong correlation with the decreased nuclear accumulation of YAP observed in the Meso-1 group (Figure 4). It is worth noting that compared to the results on day 1, the expression levels of these genes in both the Nano-1 group and the control group were significantly reduced on day 7, which was mainly due to the contact inhibition that occurred with the increased number of BMSCs.

### 3.6. The Effects of Stepped Structures on the Cell Differentiation of BMSCs

**Figure 6:**
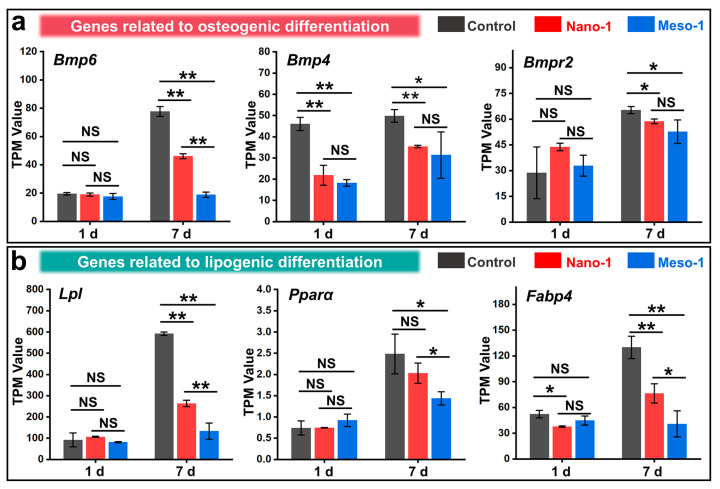
The TPM values of some genes related to (**a**) osteogenic differentiation and (**b**) lipogenic differentiation, *n* = 3. Values are expressed as mean ± SD, * *p* < 0.05, ** *p* < 0.01, and NS represents no significant difference.

Many studies have indicated that the surface structures of materials can regulate the subsequent differentiation behaviors of BMSCs by influencing their adhesions [4,56,57]. Therefore, a preliminary study on the impacts of stepped structures on the differentiation of BMSCs was conducted using RNA sequencing analysis. For the osteogenic differentiation, some gene expressions (*Bmp6*, *Bmp4*, and *Bmpr2*) of the bone morphogenetic proteins were evaluated. As shown in Figure 6a, after being cultured for 7 days, the BMSCs in the control group and Nano-1 group both exhibited a certain degree of osteogenic differentiation potential. However, the BMSCs in the Meso-1 group did not show a significant trend towards osteogenic differentiation, with the expression levels of some genes being significantly lower than those in the control group or Nano-1 group. On the other hand, the gene expressions of some adipogenic differentiation markers, such as lipoprotein lipase (*Lpl*), peroxisome proliferator-activated receptor alpha (*Pparα*), and fatty acid-binding protein 4 (*Fabp4*), were also detected. As shown in Figure 6b, after 7 days of culturing, the BMSCs in the Meso-1 group did not exhibit a significant trend toward adipogenic differentiation, with the expression levels of some genes being significantly lower than those in the control group and Nano-1 group.

These results suggested that the exposure of stepped structures on the HA surfaces not only failed to promote the positive regulation of the osteogenic and adipogenic differentiation of BMSCs but might even inhibit the differentiation capabilities of the adhered BMSCs to some extent. Based on the aforementioned changes in the physicochemical properties of the HA surfaces caused by the exposure of stepped structures (such as hydrophilicity, surface stoichiometric ratios, and the capacity for protein adsorption), and considering the impacts of stepped structures on the expression of genes related to cell adhesion and proliferation (Figure 3c and Figure 5d), it can be inferred that the stepped structures on the HA surface can affect the differentiation behaviors of BMSCs, which is likely closely related to processes such as cytoskeleton reorganization and the nuclear transfer of some mechanotransduction molecules (e.g., YAP, see Figure 4). Besides the Hippo signaling pathway, to which YAP belongs, some other signaling pathways may also play critical roles in mediating cellular responses to materials’ surface structures (Figure S9), such as the p53 signaling, focal adhesion, cell cycle, FoxO signaling, lysosome, PI3K-Akt signaling, cellular senescence, and MAPK signaling pathways.

Given that the interaction mechanism between materials’ surfaces and cells is exceedingly intricate [6,11,13], involving the extracellular–intracellular transmission of mechanical cues and the coupling of mechanical–biochemical signals, the detailed molecular mechanisms of the stepped structures regulating the functions and fate of BMSCs need to be further explored. Notwithstandingly, it is reasonable to believe that stepped structures, which contain a multitude of crystal defects (e.g., lattice vacancies) and alter geometric topographies, significantly affect various cellular behaviors of BMSCs, including adhesion, proliferation, and differentiation.

## 4. Conclusions

In summary, we have successfully prepared HA dishes with stepped structures on their surfaces and investigated their effects on the cell adhesion, proliferation, and differentiation capacities of BMSCs. The results revealed that the stepped structure altered various physicochemical properties of the HA surface, such as hydrophilicity, surface stoichiometric ratios, and protein adsorption capacity. These stepped structures were also found to significantly influence the adhesion process and morphology of BMSCs, leading to constrained cell spreading and extension, the reduced formation of filopodia and lamellipodia, decreased membrane protrusions, and fewer irregular shapes. The primary cause of this phenomenon is likely the inhibition of focal adhesion formation and maturation in BMSCs. These changes in the adhesion process and morphology had profound implications for the response process of cells to external mechanical cues. Compared to the smooth HA surfaces, the stepped structures were found to affect the cytoskeleton reorganization and nuclear translocation of YAP, indicating that the stepped structures might alter the mechanical stimuli exerted by HA surfaces on BMSCs, potentially leading to substantial effects on cell functions. Subsequent experiments also demonstrated that these stepped structures exerted varying degrees of influence on the proliferation and differentiation of BMSCs. It needs to be pointed out that this work just indicates that the stepped structures on the HA surface do not promote the positive regulation of the osteogenic and adipogenic differentiation of BMSCs; further exploration is needed regarding the effects of the stepped structures on other differentiation directions of BMSCs (such as chondrogenic differentiation and stemness retention). Therefore, this work has demonstrated that stepped structures are indeed an important characteristic of HA surfaces, shedding new light on the influence of HA surface structures on the cell behaviors of BMSCs.

Although many underlying molecular mechanisms (e.g., the transmission of mechanical cues and the coupling of mechanical–biochemical signals) and other signaling pathways (e.g., the p53 signaling pathway, focal adhesion, cell cycles) require further investigation, this work suggests that controlling surface-stepped structures could be a potential method for regulating BMSCs. For example, in some applications involving the surface modification of HA materials, stepped structures can be designed as special “cell adhesion-limiting zones” or “cell differentiation-controlling zones”. Additionally, our method for constructing stepped structures can be integrated with other processing techniques (e.g., 3D printing and ion doping), potentially opening new avenues for the material design and optimization of HA materials.

## Data Availability

The original contributions presented in the study are included in the article and Supplementary Materials, further inquiries can be directed to the corresponding authors.

## Supplementary Materials

The following supporting information can be downloaded at: https://www.mdpi.com/article/10.3390/jfb16050165/s1, Figure S1 Flowchart of the experimental method. Figure S2 XRD pattern of CaHPO_4_·2H_2_O particles. Figure S3 XRD patterns of HA mesocrystal particles, HA nanoparticles, Meso-1 sample, and Nano-1 sample. Figure S4 XPS spectra of HA mesocrystal particles, HA nanoparticles, Meso-1 sample, and Nano-1 sample: (a) full spectra, (b) C *1s* spectra, (c) O *1s* spectra, (d) Ca *2p* spectra, and (d) P *2p* spectra. Figure S5 FTIR spectrum of HA mesocrystal particles, HA nanoparticles, Meso-1 sample, and Nano-1 sample. Figure S6 (a) Flow cytometry analysis for BMSCs, *n* = 3. (b) Alizarin red staining of BMSCs before and after the osteogenic induction. (c) Fluorescence microscope images of BMSCs after the adipogenic induction (nuclei were stained blue, F-actin was stained green, PPAR-γ was stained red, scale bar = 50 μm). Figure S7 The values of roundness, circularity, and solidity of BMSCs cultured on HA dish samples for different times, *n* = 110. Values were expressed as mean ± SD, * *p* < 0.05, ** *p* < 0.01, and NS represents no significant difference. Figure S8 CCK-8 assay for the proliferation of BMSCs cultured with extracts from different HA dishes, the fresh complete culture medium was set as the control group, *n* = 6. Values were presented as mean ± SD, NS represents no significant difference. Figure S9 KEGG pathway enrichment analysis of BMSCs from the Meso-1 group versus BMSCs from the Nano-1 group.

## References

[1] Lin H., Sohn J., Shen H., Langhans M.T., Tuan R.S. (2019). Bone Marrow Mesenchymal Stem Cells: Aging and Tissue Engineering Applications to Enhance Bone Healing. Biomaterials.

[2] Pajarinen J., Lin T., Gibon E., Kohno Y., Maruyama M., Nathan K., Lu L., Yao Z., Goodman S.B. (2019). Mesenchymal Stem Cell-Macrophage Crosstalk and Bone Healing. Biomaterials.

[3] Huang G., Li F., Zhao X., Ma Y., Li Y., Lin M., Jin G., Lu T.J., Genin G.M., Xu F. (2017). Functional and Biomimetic Materials for Engineering of the Three-Dimensional Cell Microenvironment. Chem. Rev..

[4] Dalby M.J., Gadegaard N., Oreffo R.O.C. (2014). Harnessing Nanotopography and Integrin-Matrix Interactions to Influence Stem Cell Fate. Nat. Mater..

[5] Ozboyaci M., Kokh D.B., Corni S., Wade R.C. (2016). Modeling and Simulation of Protein-Surface Interactions: Achievements and Challenges. Q. Rev. Biophys..

[6] Saraswathibhatla A., Indana D., Chaudhuri O. (2023). Cell–Extracellular Matrix Mechanotransduction in 3D. Nat. Rev. Mol. Cell Biol..

[7] Zhou Y., Wu C., Chang J. (2019). Bioceramics to Regulate Stem Cells and Their Microenvironment for Tissue Regeneration. Mater. Today.

[8] Kulkarni M., Mazare A., Park J., Gongadze E., Killian M.S., Kralj S., von der Mark K., Iglič A., Schmuki P. (2016). Protein Interactions with Layers of TiO_2_ Nanotube and Nanopore Arrays: Morphology and Surface Charge Influence. Acta Biomater..

[9] Pedrosa C.R., Arl D., Grysan P., Khan I., Durrieu S., Krishnamoorthy S., Durrieu M.-C. (2019). Controlled Nanoscale Topographies for Osteogenic Differentiation of Mesenchymal Stem Cells. ACS Appl. Mater. Interfaces.

[10] Feng C., Ma B., Xu M., Zhai D., Liu Y., Xue J., Chang J., Wu C. (2020). Three-Dimensional Printing of Scaffolds with Synergistic Effects of Micro–Nano Surfaces and Hollow Channels for Bone Regeneration. ACS Biomater. Sci. Eng..

[11] Vining K.H., Mooney D.J. (2017). Mechanical Forces Direct Stem Cell Behaviour in Development and Regeneration. Nat. Rev. Mol. Cell Biol..

[12] Martino F., Perestrelo A.R., Vinarský V., Pagliari S., Forte G. (2018). Cellular Mechanotransduction: From Tension to Function. Front. Physiol..

[13] Kanchanawong P., Calderwood D.A. (2023). Organization, Dynamics and Mechanoregulation of Integrin-Mediated Cell–ECM Adhesions. Nat. Rev. Mol. Cell Biol..

[14] Kanoldt V., Kluger C., Barz C., Schweizer A.L., Ramanujam D., Windgasse L., Engelhardt S., Chrostek-Grashoff A., Grashoff C. (2020). Metavinculin modulates force transduction in cell adhesion sites. Nat. Commun..

[15] Labouesse C., Tan B.X., Agley C.C., Hofer M., Winkel A.K., Stirparo G.G., Stuart H.T., Verstreken C.M., Mulas C., Mansfield W. (2021). StemBond hydrogels control the mechanical microenvironment for pluripotent stem cells. Nat. Commun..

[16] Gegenfurtner F.A., Jahn B., Wagner H., Ziegenhain C., Enard W., Geistlinger L., Rädler J.O., Vollmar A.M., Zahler S. (2018). Micropatterning as a tool to identify regulatory triggers and kinetics of actin-mediated endothelial mechanosensing. J. Cell Sci..

[17] Strzelecka-Kiliszek A., Romiszewska M., Bozycki L., Mebarek S., Bandorowicz-Pikula J., Buchet R., Pikula S. (2019). Src and ROCK Kinases Differentially Regulate Mineralization of Human Osteosarcoma Saos-2 Cells. Int. J. Mol. Sci..

[18] Zhukova Y., Hiepen C., Knaus P., Osterland M., Prohaska S., Dunlop J.W.C., Fratzl P., Skorb E.V. (2017). The Role of Titanium Surface Nanostructuring on Preosteoblast Morphology, Adhesion, and Migration. Adv. Healthc. Mater..

[19] Lee L.C.Y., Gadegaard N., de Andrés M.C., Turner L.A., Burgess K.V., Yarwood S.J., Wells J., Salmeron-Sanchez M., Meek D., Oreffo R.O.C. (2017). Nanotopography Controls Cell Cycle Changes Involved with Skeletal Stem Cell Self-Renewal and Multipotency. Biomaterials.

[20] Koons G.L., Diba M., Mikos A.G. (2020). Materials Design for Bone-Tissue Engineering. Nat. Rev. Mater..

[21] Habraken W., Habibovic P., Epple M., Bohner M. (2016). Calcium Phosphates in Biomedical Applications: Materials for the Future?. Mater. Today.

[22] Yang C., Zhao C., Wang X., Shi M., Zhu Y., Jing L., Wu C., Chang J. (2019). Stimulation of Osteogenesis and Angiogenesis by Micro/Nano Hierarchical Hydroxyapatite: Via Macrophage Immunomodulation. Nanoscale.

[23] Zhao C., Xia L., Zhai D., Zhang N., Liu J., Fang B., Chang J., Lin K. (2015). Designing Ordered Micropatterned Hydroxyapatite Bioceramics to Promote the Growth and Osteogenic Differentiation of Bone Marrow Stromal Cells. J. Mater. Chem. B.

[24] Yang W., Han W., He W., Li J., Wang J., Feng H., Qian Y. (2016). Surface Topography of Hydroxyapatite Promotes Osteogenic Differentiation of Human Bone Marrow Mesenchymal Stem Cells. Mater. Sci. Eng. C.

[25] Nasiri N., Mukherjee S., Panneerselvan A., Nisbet D.R., Tricoli A. (2018). Optimally Hierarchical Nanostructured Hydroxyapatite Coatings for Superior Prosthesis Biointegration. ACS Appl. Mater. Interfaces.

[26] Nada H. (2014). Difference in the Conformation and Dynamics of Aspartic Acid on the Flat Regions, Step Edges, and Kinks of a Calcite Surface: A Molecular Dynamics Study. J. Phys. Chem. C.

[27] Liao C., Zhou J. (2014). Replica-Exchange Molecular Dynamics Simulation of Basic Fibroblast Growth Factor Adsorption on Hydroxyapatite. J. Phys. Chem. B.

[28] Friddle R.W., Battle K., Trubetskoy V., Tao J., Salter E.A., Moradian-Oldak J., De Yoreo J.J., Wierzbicki A. (2011). Single-Molecule Determination of the Face-Specific Adsorption of Amelogenin’s C-Terminus on Hydroxyapatite. Angew. Chem..

[29] Makrodimitris K., Masica D.L., Kim E.T., Gray J.J. (2007). Structure Prediction of Protein-Solid Surface Interactions Reveals a Molecular Recognition Motif of Statherin for Hydroxyapatite. J. Am. Chem. Soc..

[30] Wu C., Chen M., Xing C. (2010). Molecular Understanding of Conformational Dynamics of a Fibronectin Module on Rutile (110) Surface. Langmuir.

[31] Lin K., Xia L., Gan J., Zhang Z., Chen H., Jiang X., Chang J. (2013). Tailoring the Nanostructured Surfaces of Hydroxyapatite Bioceramics to Promote Protein Adsorption, Osteoblast Growth, and Osteogenic Differentiation. ACS Appl. Mater. Interfaces.

[32] Liao C., Xie Y., Zhou J. (2014). Computer Simulations of Fibronectin Adsorption on Hydroxyapatite Surfaces. RSC Adv..

[33] De Yoreo J.J., Gilbert P.U.P.A., Sommerdijk N.A.J.M., Penn R.L., Whitelam S., Joester D., Zhang H., Rimer J.D., Navrotsky A., Banfield J.F. (2015). Crystallization by Particle Attachment in Synthetic, Biogenic, and Geologic Environments. Science.

[34] Dandekar P., Kuvadia Z.B., Doherty M.F. (2013). Engineering Crystal Morphology. Annu. Rev. Mater. Res..

[35] Chen J., Lei B., Xie H., Zhou L., Wang S. (2017). Easy Way to Create Stepped Surface: A Thought from Oriented Attachment. Chem. Mater..

[36] Chen J., Huang Z., Wang F., Gong M., Zhang X., Wang Y., Hu Z., Zeng Z., Wang Y. (2022). The Restricted Adhesion of Bone Marrow Mesenchymal Stem Cells by Stepped Structures on Surfaces of Hydroxyapatite. RSC Adv..

[37] Ohka T., Akiyama T., Pradipto A.M., Nakamura K., Ito T. (2020). Effect of Step Edges on Adsorption Behavior for GaN(0001) Surfaces during Metalorganic Vapor Phase Epitaxy: An Ab Initio Study. Cryst. Growth Des..

[38] Yin X., Geng D., Wang X. (2016). Inverted Wedding Cake Growth Operated by the Ehrlich-Schwoebel Barrier in Two-Dimensional Nanocrystal Evolution. Angew. Chem. Int. Ed..

[39] Morin S.A., Forticaux A., Bierman M.J., Jin S. (2011). Screw Dislocation-Driven Growth of Two-Dimensional Nanoplates. Nano Lett..

[40] Yin X., Shi J., Niu X., Huang H., Wang X. (2015). Wedding Cake Growth Mechanism in One-Dimensional and Two-Dimensional Nanostructure Evolution. Nano Lett..

[41] Dai J., Lu J., Wang F., Guo J., Gu N., Xu C. (2015). Optical and Exciton Dynamical Properties of a Screw-Dislocation-Driven ZnO:Sn Microstructure. ACS Appl. Mater. Interfaces.

[42] Zou Z., Liu X., Chen L., Lin K., Chang J. (2012). Dental Enamel-like Hydroxyapatite Transformed Directly from Monetite. J. Mater. Chem..

[43] Chen J., Li X., Lei B., Zhou L., Wang S. (2019). Visible Light Photocatalysis of Pristine Anatase TiO_2_ Mesocrystals Induced by Largely Exposed and Stepped {001} Surface. Green. Chem..

[44] Surmeneva M.A., Surmenev R.A., Tyurin A.I., Mukhametkaliyev T.M., Teresov A.D., Koval N.N., Pirozhkova T.S., Shuvarin I.A., Oehr C. (2014). Comparative Study of the Radio-Frequency Magnetron Sputter Deposited CaP Films Fabricated onto Acid-Etched or Pulsed Electron Beam-Treated Titanium. Thin Solid. Film..

[45] Saroj S., Vijayalakshmi U. (2025). Structural, morphological and biological assessment of magnetic hydroxyapatite with superior hyperthermia potential for orthopedic applications. Sci. Rep..

[46] Wu X., Zhou M., Jiang F., Yin S., Lin S., Yang G., Lu Y., Zhang W., Jiang X. (2021). Marginal Sealing around Integral Bilayer Scaffolds for Repairing Osteochondral Defects Based on Photocurable Silk Hydrogels. Bioact. Mater..

[47] Uynuk-Ool T., Rothdiener M., Walters B., Hegemann M., Palm J., Nguyen P., Seeger T., Stöckle U., Stegemann J.P., Aicher W.K. (2017). The Geometrical Shape of Mesenchymal Stromal Cells Measured by Quantitative Shape Descriptors Is Determined by the Stiffness of the Biomaterial and by Cyclic Tensile Forces. J. Tissue Eng. Regen. Med..

[48] Amann E., Amirall A., Franco A.R., Poh P.S.P., Sola Dueñas F.J., Fuentes Estévez G., Leonor I.B., Reis R.L., van Griensven M., Balmayor E.R. (2021). A Graded, Porous Composite of Natural Biopolymers and Octacalcium Phosphate Guides Osteochondral Differentiation of Stem Cells. Adv. Healthc. Mater..

[49] Chen Y., Zhai M.J., Mehwish N., Xu M.D., Wang Y., Gong Y.X., Ren M.M., Deng H., Lee B.H. (2022). Comparison of Globular Albumin Methacryloyl and Random-Coil Gelatin Methacryloyl: Preparation, Hydrogel Properties, Cell Behaviors, and Mineralization. Int. J. Biol. Macromol..

[50] Özkale B., Sakar M.S., Mooney D.J. (2021). Active Biomaterials for Mechanobiology. Biomaterials.

[51] Chandorkar Y., Castro Nava A., Schweizerhof S., Van Dongen M., Haraszti T., Köhler J., Zhang H., Windoffer R., Mourran A., Möller M. (2019). Cellular Responses to Beating Hydrogels to Investigate Mechanotransduction. Nat. Commun..

[52] Liu Z., Wang L., Xu H., Du Q., Li L., Wang L., Zhang E.S., Chen G., Wang Y. (2020). Heterogeneous Responses to Mechanical Force of Prostate Cancer Cells Inducing Different Metastasis Patterns. Adv. Sci..

[53] Kalukula Y., Stephens A.D., Lammerding J., Gabriele S. (2022). Mechanics and Functional Consequences of Nuclear Deformations. Nat. Rev. Mol. Cell Biol..

[54] Hyväri L., Vanhatupa S., Halonen H.T., Kääriäinen M., Miettinen S. (2020). Myocardin-Related Transcription Factor A (MRTF-A) Regulates the Balance between Adipogenesis and Osteogenesis of Human Adipose Stem Cells. Stem Cells Int..

[55] Ferreira S.A., Faull P.A., Seymour A.J., Yu T.T.L., Loaiza S., Auner H.W., Snijders A.P., Gentleman E. (2018). Neighboring Cells Override 3D Hydrogel Matrix Cues to Drive Human MSC Quiescence. Biomaterials.

[56] Zhao C., Wang X., Gao L., Jing L., Zhou Q., Chang J. (2018). The Role of the Micro-Pattern and Nano-Topography of Hydroxyapatite Bioceramics on Stimulating Osteogenic Differentiation of Mesenchymal Stem Cells. Acta Biomater..

[57] Chen Z., Bachhuka A., Wei F., Wang X., Liu G., Vasilev K., Xiao Y. (2017). Nanotopography-Based Strategy for the Precise Manipulation of Osteoimmunomodulation in Bone Regeneration. Nanoscale.

