# Supplementary material for: Engineering Stepped Structures on Hydroxyapatite Surfaces: A Potential Strategy to Modulate Bone Marrow Mesenchymal Stem Adhesion, Spreading, and Proliferation
Source: J Funct Biomater. 2025 May 8;16(5):165. doi: 10.3390/jfb16050165 (PMC12112300; doi:10.3390/jfb16050165)
Supplement: Supplementary file 1 [file jfb-16-00165-s001.zip › jfb-3579460-supplementary.pdf]

Engineering stepped structures on hydroxyapatite surfaces: a potential strategy to modulate bone marrow mesenchymal stem adhesion, spreading, and proliferation

Yongmei Wang [a,#], Fang Wang [a,#], Min Gong [a], Lidan Chen [b], Yun Wang [a], Pu Xu [b], Zhu Zeng [a,b,*], Zuquan Hu [a,b], Jin Chen [a,b,*]

[a] Key Laboratory of Infectious Immune and Antibody Engineering of Guizhou Province, School of Basic Medical Sciences, Guizhou Medical University, Guiyang, 561113, P. R. China.

[b] Key Laboratory of Biology and Medical Engineering/Immune Cells and Antibody Engineering Research Center of Guizhou Province, School of Biology and Engineering, Guizhou Medical University, Guiyang, 561113, P. R. China.

[#] These authors contributed equally to this work.

[*] Corresponding Author.

E-mail: zengzhu@gmc.edu.cn; chenjin_fdt@163.com;

Tel: +86-0851-88174044

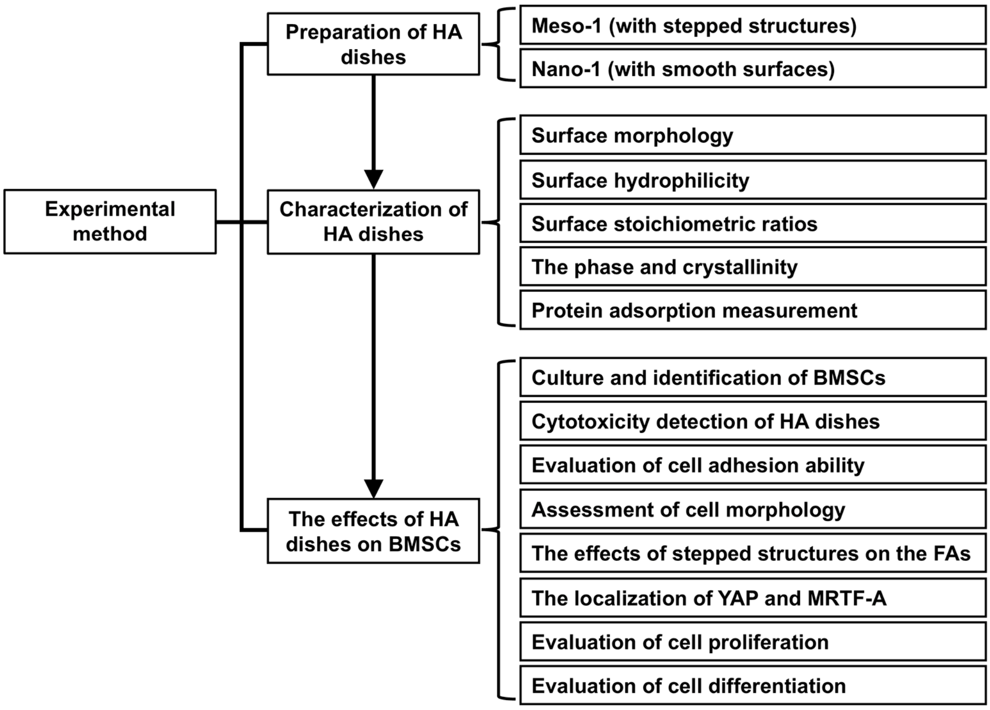

Figure S1 Flowchart of the experimental method.

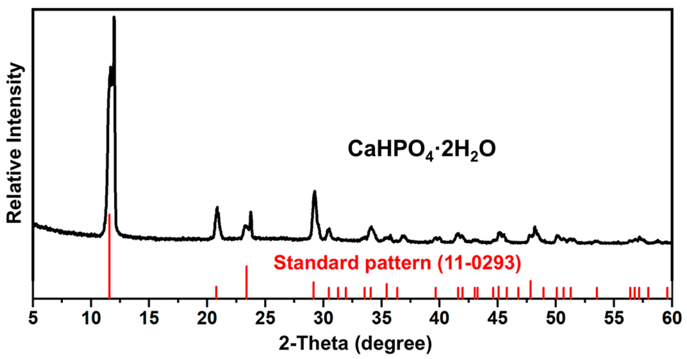

Figure S2 XRD pattern of $CaHPO_4 \cdot 2H_2O$ particles.

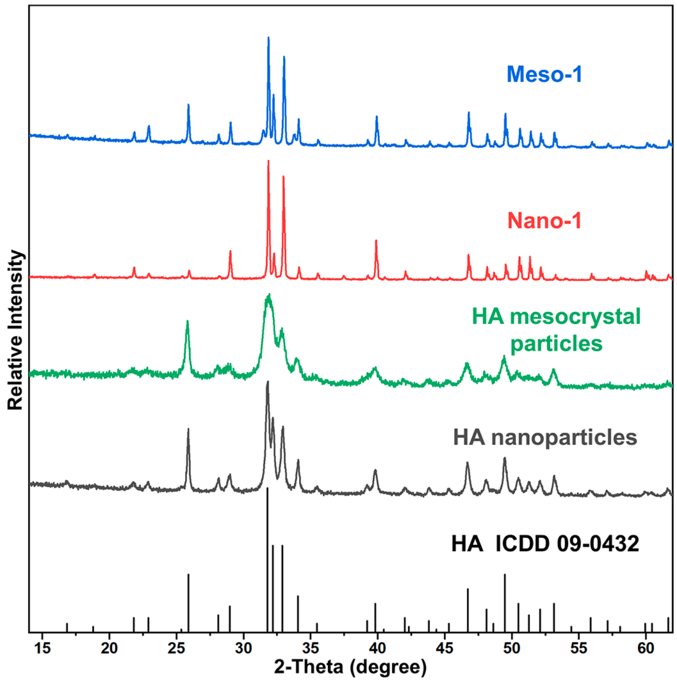

Figure S3 XRD patterns of HA mesocrystal particles, HA nanoparticles, Meso-1 sample, and Nano-1 sample.

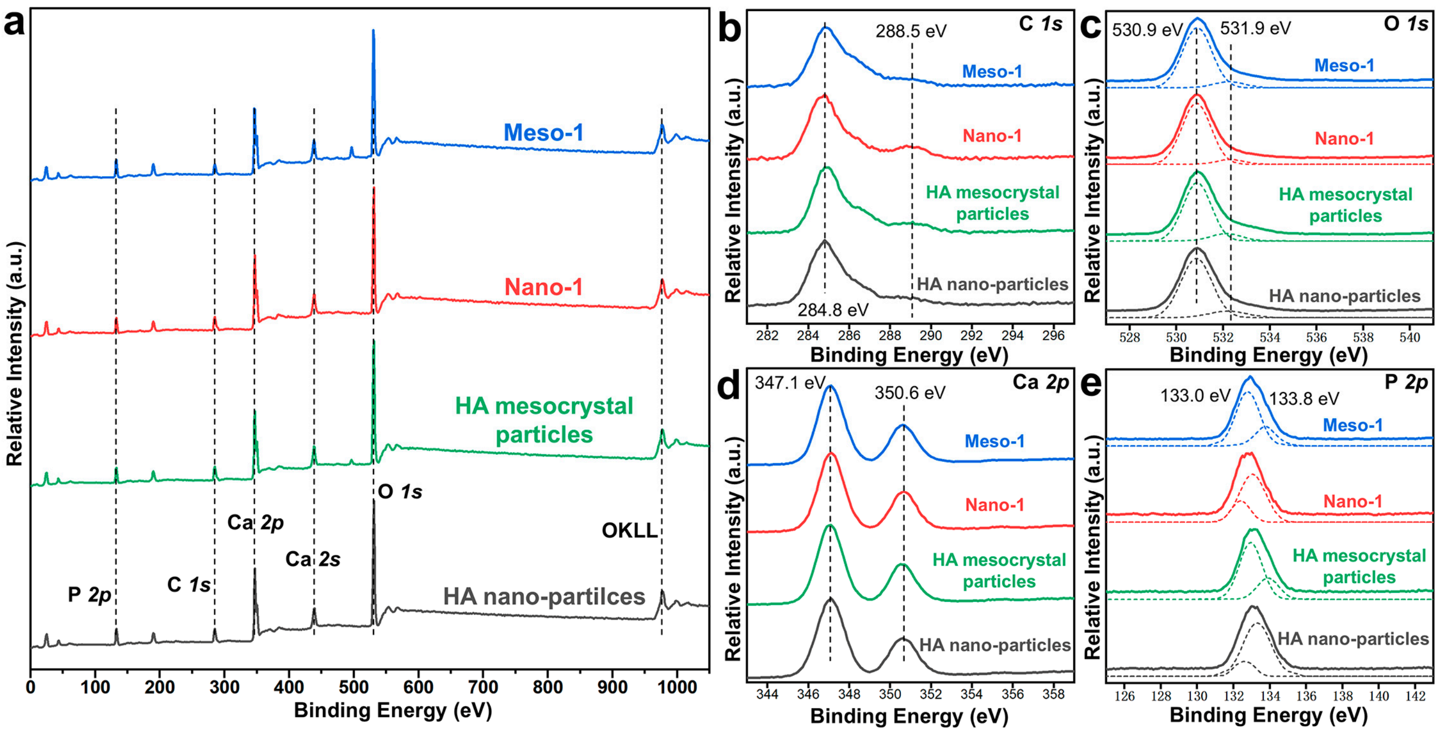

Figure S4 XPS spectra of HA mesocrystal particles, HA nanoparticles, Meso-1 sample, and Nano-1 sample: (a) full spectra, (b) C *1s* spectra, (c) O *1s* spectra, (d) Ca *2p* spectra, and (d) P *2p* spectra.

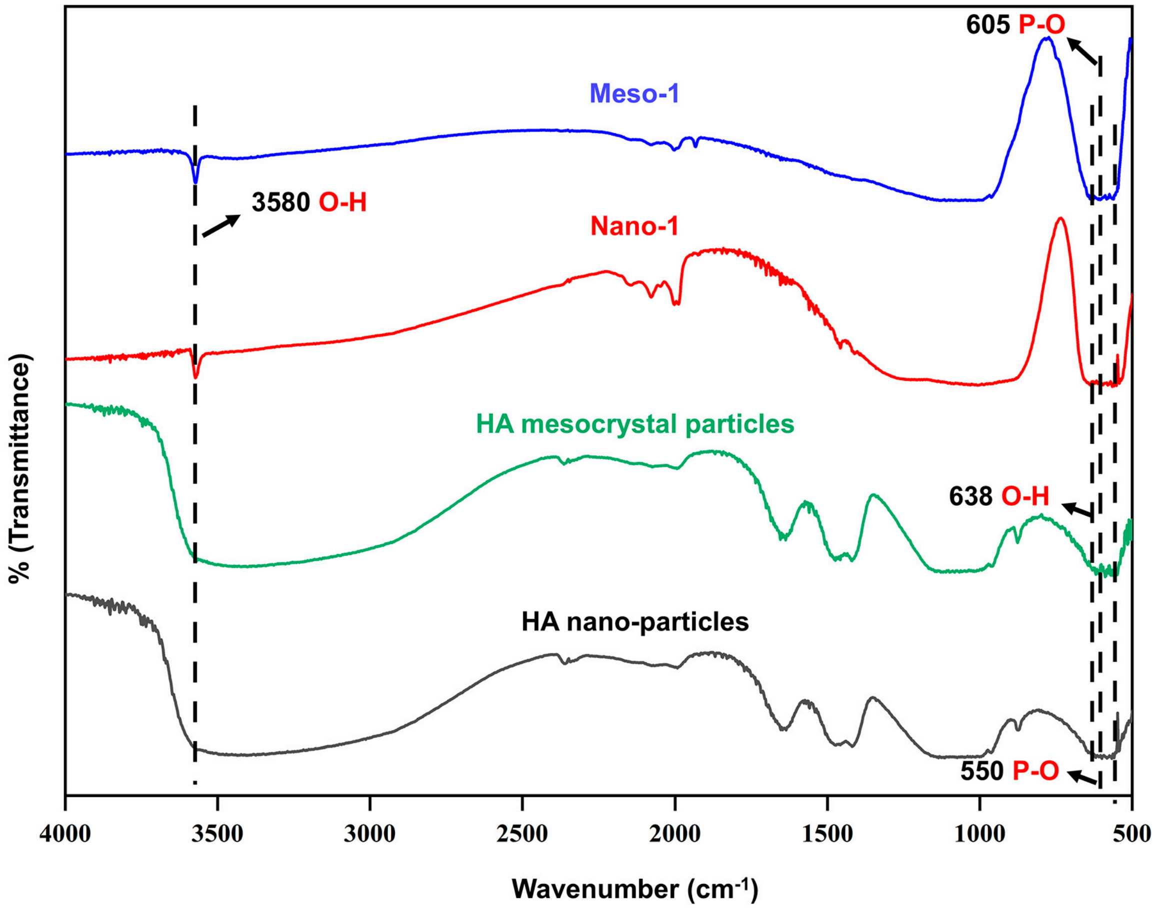

Figure S5 FTIR spectra of HA mesocrystal particles, HA nanoparticles, Meso-1 sample, and Nano-1 sample.

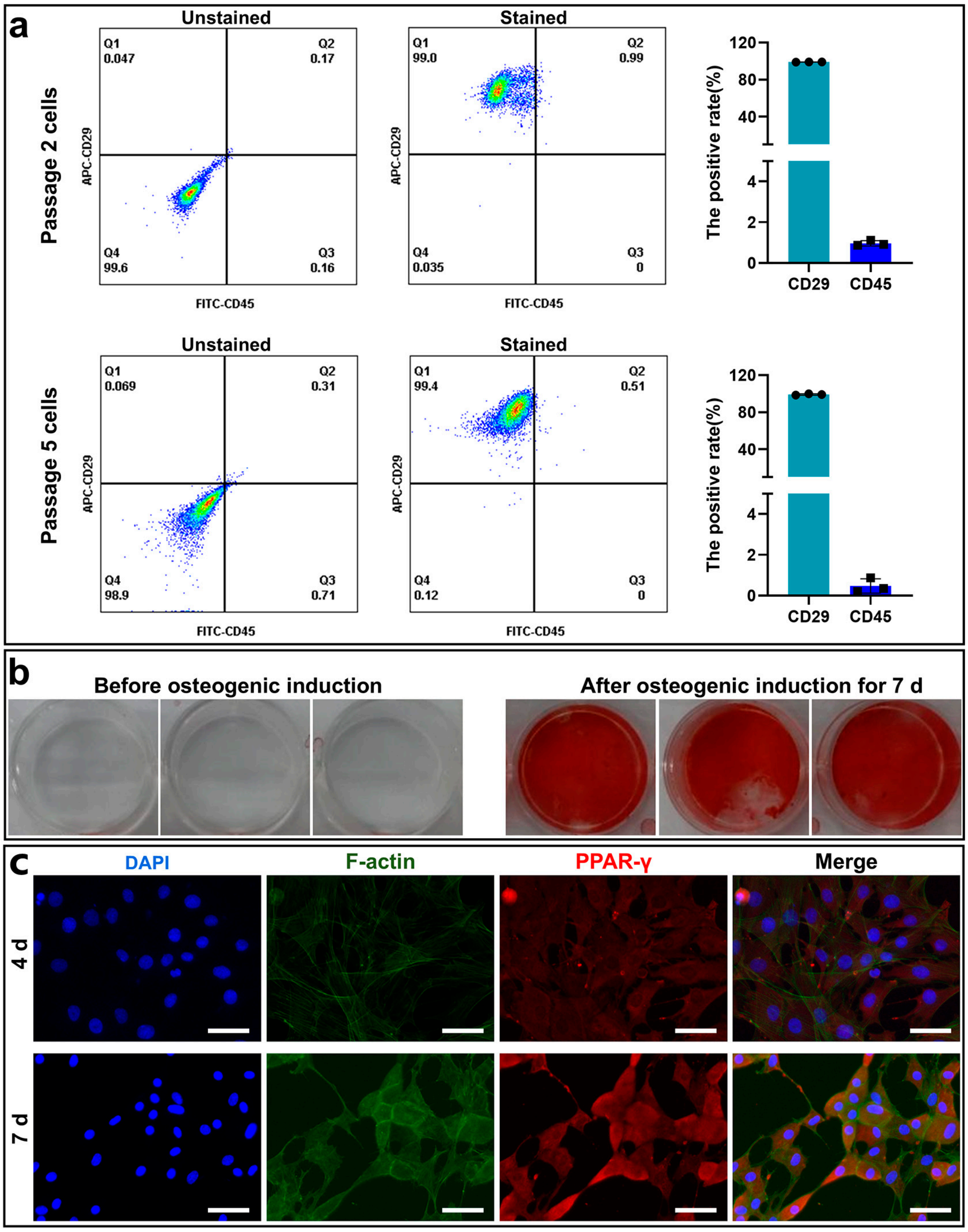

Figure S6 (a) Flow cytometry analysis for BMSCs, n = 3. (b) Alizarin red staining of BMSCs before and after the osteogenic induction. (c) Fluorescence microscope images of BMSCs after the adipogenic induction (nuclei were stained blue, F-actin was stained green, PPAR-γ was stained red, scale bar = 50 μm).

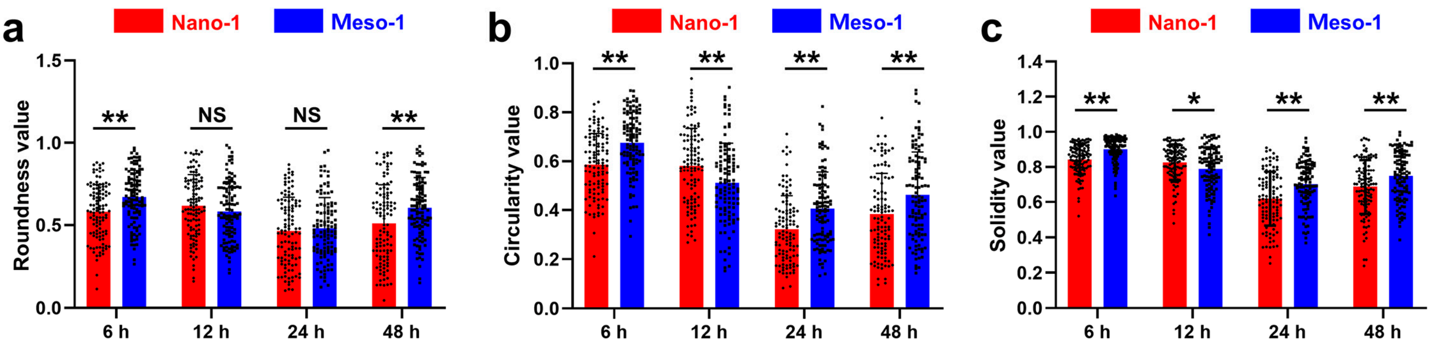

Figure S7 The values of roundness, circularity, and solidity of BMSCs cultured on HA dish samples for different times, n=110. Values were expressed as mean ± SD, *$P < 0.05$, **$P < 0.01$, and NS represents no significant difference.

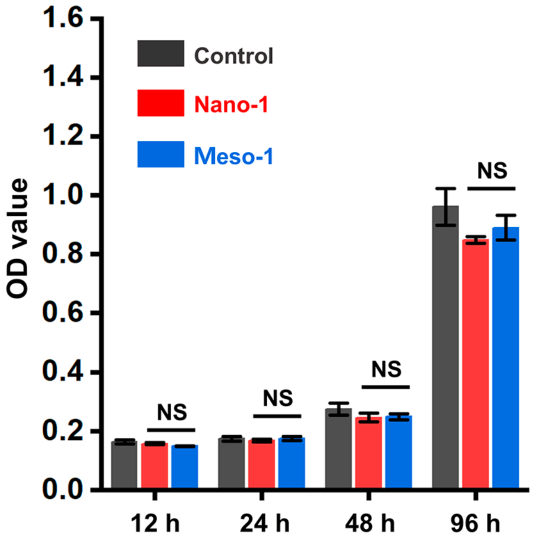

Figure S8 CCK-8 assay for the proliferation of BMSCs cultured with extracts from different HA dishes, the fresh complete culture medium was set as the control group, n = 6. Values were presented as mean ± SD, NS represents no significant difference.

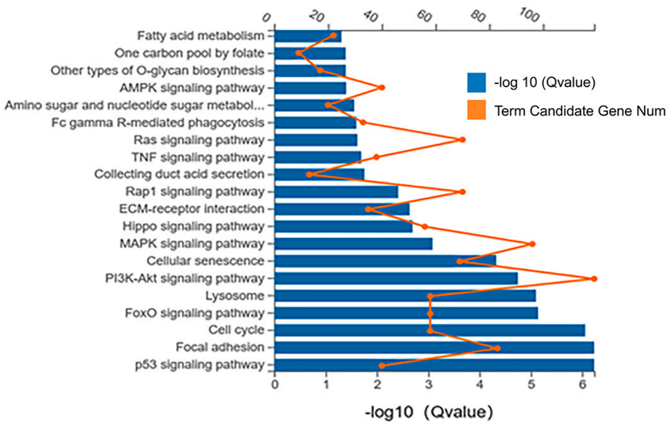

Figure S9 KEGG pathway enrichment analysis of BMSCs from the Meso-1 group versus BMSCs from the Nano-1 group.
